# Goats prefer positive human emotional facial expressions

**DOI:** 10.1098/rsos.180491

**Published:** 2018-08-29

**Authors:** Christian Nawroth, Natalia Albuquerque, Carine Savalli, Marie-Sophie Single, Alan G. McElligott

**Affiliations:** 1 Biological and Experimental Psychology, School of Biological and Chemical Sciences, Queen Mary University of London, London, UK; 2 Institute of Behavioural Physiology, Leibniz Institute for Farm Animal Biology, Dummerstorf, Germany; 3 Federal Food Safety and Veterinary Office FSVO, Centre for Proper Housing of Ruminants and Pigs, Agroscope Tänikon, Ettenhausen, Switzerland; 4 Department of Experimental Psychology, Institute of Psychology, University of Sao Paulo, Sao Paulo, Brazil; 5 Department of Public Politics and Public Health, Federal University of Sao Paulo, Santos, Brazil; 6 Physiology Weihenstephan, Technical University of Munich, Freising, Germany; 7 Centre for Research in Ecology, Evolution and Behaviour, Department of Life Sciences, University of Roehampton, London, UK

**Keywords:** emotions, emotion perception, interspecific communication, livestock, social cognition

## Abstract

Domestication has shaped the physiology and the behaviour of animals to better adapt to human environments. Therefore, human facial expressions may be highly informative for animals domesticated for working closely with people, such as dogs and horses. However, it is not known whether other animals, and particularly those domesticated primarily for production, such as goats, are capable of perceiving human emotional cues. In this study, we investigated whether goats can distinguish human facial expressions when simultaneously shown two images of an unfamiliar human with different emotional valences (positive/happy or negative/angry). Both images were vertically attached to a wall on one side of a test arena, 1.3 m apart, and goats were released from the opposite side of the arena (distance of 4.0 m) and were free to explore and interact with the stimuli during the trials. Each of four test trials lasted 30 s. Overall, we found that goats preferred to interact first with happy faces, meaning that they are sensitive to human facial emotional cues. Goats interacted first, more often and for longer duration with positive faces when they were positioned on the right side. However, no preference was found when the positive faces were placed on the left side. We show that animals domesticated for production can discriminate human facial expressions with different emotional valences and prefer to interact with positive ones. Therefore, the impact of domestication on animal cognitive abilities may be more far-reaching than previously assumed.

## 1. Introduction

Facial expressions are rich sources of social information for humans and thus have an important role in regulating social interactions [1–3]. In addition, facial expressions are also prevalent in non-human animals [4,5], and the question of whether and how animals perceive emotional facial expressions is of major interest to understand their underlying ultimate functions and phylogenetic origins [6,7].

Humans and non-human primates possess a rich repertoire of facial expressions [8] and are capable of discriminating emotional facial expressions of conspecifics [2,9–11]. Although primate musculature facilitates communication through facial cues, non-primate species that possess less developed facial muscles might also be able to convey information to others through their faces [5,12,13]. Sheep (*Ovis aries*), for example, have shown some sophisticated social skills, including long-term visual memory of conspecific faces [14,15] and discrimination of images of conspecifics expressing different emotions [16]. Individual discrimination of heterospecifics is present in non-human animals such as birds [17,18], horses [19] and dogs [20]. However, discriminating facial expressions linked to emotions in heterospecifics, such as humans, is assumed to be particularly challenging because emotions are not necessarily expressed in similar ways across species [4].

As a result of their domestication as companion animals, dogs (*Canis familiaris*) are very good at perceiving human communicative cues [21,22]. Dogs are able to discriminate human emotional facial expressions [23] and can categorize and integrate visual and acoustic emotional information of different valences [24]. Recently, horses (*Equus caballus*) have also been found to react with functionally relevant responses to human faces of different emotional valences [25] and to remember emotional facial expressions of individual humans [26]. It has been suggested that this ability of processing heterospecific emotional expressions is a by-product of their close working relationships with humans during domestication [27]. Although the domestication history of dogs and horses differs, both were domesticated to cooperate with humans in a variety of contexts, such as for hunting, guarding or riding. In these cooperative contexts, the perception of human emotional facial expressions is likely to have been adaptive for both species.

Unlike dogs and horses, goats have been exclusively domesticated for production of materials used by humans [28,29], with new findings indicating that early intentional human efforts for selecting these animals (dating 8000 years) were focused on pigmentation, stature, reproduction, milking and response to dietary change [30]. These differences may suggest that domestic goats are less likely to have been selected for reading subtle communicative cues from humans. However, an initial selection for tameness and a thus reduced emotional reactivity might have been sufficient to enhance a general human–animal communication set of skills in domestic animals [31]. In agreement with the latter idea, recent research has found that goats are sensitive to more salient human behaviours (e.g. express human-directed behaviours during problem-solving tasks [32,33] and alter their behaviour depending on human attentive state [34]). These results challenge the idea that such socio-cognitive adaptations are limited to companion or working animals, such as dogs and horses, and therefore it is possible that goats may also possess the ability to perceive more subtle communicative cues, such as human facial expressions that are linked to emotions.

To determine whether the sensitivity to human emotional expressions can be found in animals apart from domestic companion or working species, we investigated whether goats can distinguish human facial expressions with different emotional valences. We hypothesized that goats, a domestic species not selected for working closely with humans, are able to differentiate between human emotional facial expressions. We also expected goats to prefer to approach positive human facial expressions compared to negative ones.

## 2. Material and methods

### 2.1. Subjects and housing

The study was carried out at a goat sanctuary (Buttercups Sanctuary for Goats, http://www.buttercups.org.uk) in the UK. Initially, a total of 35 goats were tested (14 females and 21 castrated males), which were fully habituated to human presence because of previous research [33,35]. Goats were excluded if they did not pass the training phase (i.e. they had to approach the experimenter within 30 s; *n* = 5) or did not gaze/interact with the images in the first trial (*n* = 10), indicating a lack of interest to interact with the images. Thus, 20 subjects (eight females and 12 males) received a total of four test trials each. These goats (various breeds) were aged 3–19 years (electronic supplementary material, table S1). Routine care of the animals was provided by sanctuary employees and volunteers. The goats had ad libitum access to hay and were not food-restricted before testing.

### 2.2. Stimuli

**Figure 1.**
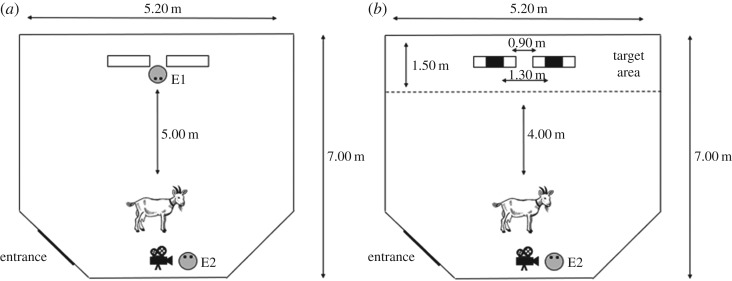
Set-up of the task for training (*a*) and test trials (*b*).

We presented the goats with pairs of greyscale still human faces of the same individual (either female or male) showing positive (happy) and negative (angry) facial expressions (human stimuli used in [24]). The faces were printed on white A3 paper and placed onto a square metal mesh (100 × 125 cm) at a height of approximately 60 cm. Both meshes were positioned 90 cm apart from each other in training and test trials (figure 1). The models in the pictures (one woman and one man) were unfamiliar to all subjects. We used two-dimensional (2D) images rather than live human presentations to control for potential subtle cues and variation in the expressions displayed. Indeed, 2D images of conspecifics have successfully been used in previous studies of ungulate visual perception (e.g. [13,28,29]).

### 2.3. General procedure

The experiment was carried out in a temporary enclosure made of metal hurdles (700 × 520 cm, figure 1), which was set up within the normal daytime range of the goats. Subjects were tested from 12.00 to 16.00 from August to October 2016. The test subjects were brought to the experimental arena placed in the field. They were visually isolated from other goats but remained in auditory and olfactory contact with them.

#### 2.3.1. Training

Experimenter 1 (E1) was positioned between two metal hurdles at the end of the test arena, visibly holding one piece of dry pasta in each hand (figure 1). E1 remained still, showed a neutral facial expression and looked down towards the ground. A small food reward was used in training to motivate subjects to explore the opposite side of the arena during the subsequent test trials. The human facial images were attached to the metal hurdles but turned around so that they were not visible to the subjects during training. Experimenter 2 (E2) held the goat on a lead on the other side of the arena, in the middle of the area and 5 m away from E1 (figure 1). E2 was standing next to the goat, and the side which the goat was held from was randomized to avoid cueing. After E1 took up position, E2 released the goat, which could then approach E1 and get the food. Subjects received three training trials (before every testing session) and had to approach E1 in all three trials to proceed to the test. Training trials were terminated when a subject did not approach the other side of the arena, i.e. the experimenter with the food reward, within 30 s. E2 remained still and showed a neutral facial expression as well as a constant gaze directed behind the arena during training and test trials.

#### 2.3.2. Test

Immediately after training, the subject goat was brought back to the starting point and while E2 distracted the test subject, E1 turned the metal hurdles with the stimuli attached, making them now visible. After turning the hurdles, E1 left the arena via an exit behind the test subject (figure 1) and went out of sight to avoid distracting the animal. E2 then turned the goat towards the stimuli and released it. Again, the side on which E2 held the goat was randomized. The subject was free to move around the arena and approach and interact with the two images. Each subject was tested in a series of four experimental sessions; each consisted of three training trials and one test trial and were two weeks apart from each other (i.e. testing occurred over a total of eight weeks). For each test trial, half of the subjects received the male faces, while the other subjects received the female ones. In addition, for each test trial, half of the subjects were presented with the positive human face on the left side first, and the other half with the positive human face on the right side first. The gender of the stimuli and the side of presentation were counter-balanced within and between subjects. Thus, there were four possible stimuli combinations, presented in a randomized order: happy woman on left + angry woman on the right; happy woman on the right + angry woman on the left; happy man on the left + angry man on the right; happy man on the right + angry man on the left. All subjects saw all combinations.

### 2.4. Data coding and analysis

All trials were videotaped (Sony HCR-CX190E Camcorder) and analysed using a Simple Video Coder [36]. The test trial started as soon as a goat entered the target area close to the images (figure 1) and lasted for 30 s. The time subjects spent between the start point and starting the test varied (up to 30 s). During the test trial, we recorded the direction of the first interaction (towards the positive or negative face) as well as subjects' rate and the duration of interactions with both images. Interactions were considered as gazing towards or physically interacting with the images. Gazing was defined as being positioned in the target area and having their head oriented towards one of the images. Physical interaction was defined as touching the image with their snout. If subjects did not enter the target area after 30 s, the trial was terminated. A second coder, unfamiliar with the hypothesis, scored the subjects’ interactions with the images for 25% of the total trials. Inter-observer agreement for duration (Kendall's concordance coefficient: positive image: W = 0.974, *p* = 0.008; negative image: W = 0.942, *p* = 0.011) and rate (Kendall's concordance coefficient: positive image: W = 0.849, *p* = 0.029; negative image: W = 0.909, *p* = 0.016) was high, indicating that interactions with the positive and negative image could be unambiguously classified.

For the duration and rate of interactions, we calculated an emotional valence index [(*P* − *N*)/(*P* + *N*)], where *P* and *N* represent the amount of time the goat interacted with the positive (*P*) and negative image (*N*). This index resulted in values of ‘−1’ or ‘1’ for almost 70% of our data, suggesting that when goats approached an image (positive or negative), they tended to remain close to that image's side, which made the distribution of duration and rate bimodal. For this reason, these raw variables were categorized, and transformed into binary data, as follows: positive values indicate a preference for the positive face and negative values indicate a preference for the negative face. Trials where goats did not make any choice were excluded from the analyses because they were not informative for the interpretation of the data (13 trials distributed among 10 individuals, representing 16.2% of all trials). First interaction (positive or negative image) and the indices for duration and rate were analysed using a generalized estimated equation (GEE) model for binary data with logit link function; the within-subjects dependence was incorporated by using an exchangeable structure, which assumes same correlation among measures taken from the same individual (thus controlling for pseudo-replication). For each response variable, we tested at first whether there was a difference for positive and negative images and, in a second model, we tested whether three fixed effects, i.e. the side of the positive face (left versus right), gender of the stimulus (male or female) and gender of the subject (male or female), as well as their first-order (two-by-two) interactions, influenced our goats’ behaviour. Additionally, in separate models, we also tested for a general side bias (left or right side of the arena) for first interaction, and categorized rate and duration using GEE models. The significance level was set to 0.05. All analysis was conducted with SAS software v. 9.2 (SAS Institute, Inc., Cary, NC, USA).

## 3. Results

### 3.1. First interaction

**Figure 2:**
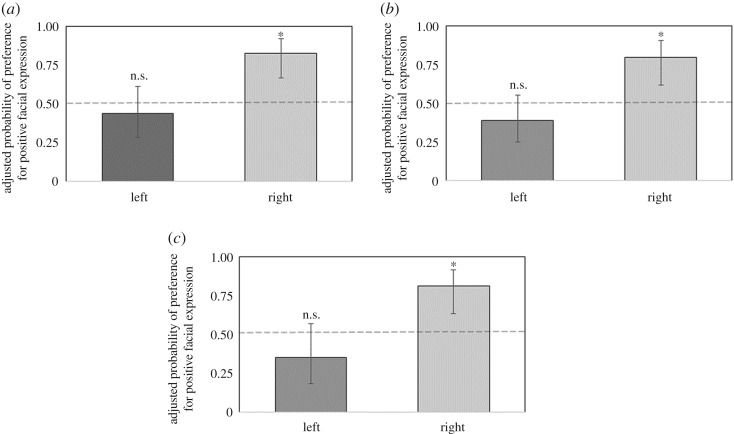
Adjusted probabilities of preference for positive facial expression for first interaction (*a*), duration (*b*) and rate (*c*). Asterisks indicate significant deviation from chance level. n.s.: no significant deviation.

Considering all trials, goats' first interactions were more often with the positive image (Wald = 6.66, d.f. = 1, *p* = 0.0098). The second model showed no two-by-two interaction between the three factors analysed (human gender * sex of the goats: Wald = 0.22, d.f. = 1, *p* = 0.64; human gender * side of positive image: Wald = 1.12, d.f. = 1, *p* = 0.29; sex of the goats * side of positive image: Wald = 0.59, d.f. = 1, *p* = 0.44); therefore, we adjusted the model including just main factors. We found no main effect of human gender, or sex of the goats on the probability of interacting first with positive images (human gender: Wald = 0.03; d.f. = 1, *p* = 0.87; sex of the goats: Wald = 0.02; d.f. = 1, *p* = 0.90). However, the side on which the positive image was placed (left or right) significantly affected the goats’ first interaction (Wald = 8.90; d.f. = 1, *p* = 0.0029). The adjusted probability of interacting first with the positive image was 0.82 (CL95%: 0.67–0.92) when it was placed on the right side (Wald = 12.73, d.f. = 1, *p* = 0.0004, figure 2), whereas it was 0.44 (CL95%: 0.28–0.61) when it was placed on the left side (Wald = 0.49, d.f. = 1, *p* = 0.49, figure 2). Finally, we found a general side bias; goats were more likely to interact first with images on the right side of the arena compared to those on the left (Wald = 9.63, d.f. = 1, *p* = 0.0019).

### 3.2. Duration of interactions

Considering all trials, goats tended to spend more time with the positive image compared to the negative one, i.e. the probability of spending more time with positive images tended to be greater than 0.5 (Wald = 3.73, d.f. = 1, *p* = 0.0533). The second model showed no two-by-two interaction between the three factors analysed (human gender * sex of the goats: Wald = 0.07, d.f. = 1, *p* = 0.79; human gender * side of positive image: Wald = 0.37, d.f. = 1, *p* = 0.54; sex of the goats * side of positive image: Wald = 0.60, d.f. = 1, *p* = 0.44); therefore, we adjusted the model including just main factors. We found no main effect of human gender, or sex of the goats tested on the probability of spending more time with positive images (human gender: Wald = 0.01; d.f. = 1, *p* = 0.93; sex of the goats: Wald = 0.50; d.f. = 1, *p* = 0.48). However, the side on which the positive image was placed (left or right) also affected the duration of interaction with the images (Wald = 8.24; d.f. = 1, *p* = 0.0041). The adjusted probability of spending more time with the positive image was 0.79 (CL95%: 0.62–0.90) when it was placed on the right side (Wald = 9.08, d.f. = 1, *p* = 0.0026, figure 2), whereas it was 0.40 (CL95%: 0.25–0.55) when placed on the left side (Wald = 1.79, d.f. = 1, *p* = 0.18, figure 2). Finally, we found a general side bias; goats were more likely to interact longer with images on the right side rather than the left side of the arena (Wald = 7.67, d.f. = 1, *p* = 0.0056).

### 3.3. Rate of interactions

Considering all trials, goats did not show a general tendency to interact more often with either the positive or the negative image, i.e. the probability of interacting with positive images is not different from 0.5 (Wald = 1.46, d.f. = 1, *p* = 0.2264). The second model showed no two-by-two interaction between the three factors analysed (human gender * sex of the goats: Wald = 0.73, d.f. = 1, *p* = 0.39; human gender * side of positive image: Wald = 0.51, d.f. = 1, *p* = 0.47; sex of the goats * side of positive image: Wald = 2.30, d.f. = 1, *p* = 0.13); therefore, we adjusted the model including only the main factors. We found no main effect of human gender of the images, or sex of the goats tested, on the probability to interact more often with the images (human gender: Wald = 0.12; d.f. = 1, *p* = 0.73; sex of the goats: Wald = 0.54, d.f. = 1, *p* = 0.46). However, the side on which the positive image was placed (left or right) affected goats' rate of interaction with the images (Wald = 8.01, d.f. = 1, *p* = 0.0047). The adjusted probability of interacting more often with the positive image was 0.81 (CL95%: 0.63–0.91) when it was placed on the right side (Wald = 0.91, d.f. = 1, *p* = 0.0018, figure 2), whereas it was 0.35 (CL95%: 0.18–0.57) when it was placed on the left side (Wald = 1.85, d.f. = 1, *p* = 0.17, figure 2). Finally, we again found a general side bias; goats were more likely to interact more often with images on the right side rather than the left side of the arena (Wald = 10.47, d.f. = 1, *p* = 0.0012).

## 4. Discussion

Faces are some of the most important and salient classes of stimuli involved in social communication for both human and non-human animals [6,37]. In the case of domestic companion animals, both conspecific and heterospecific facial expressions are informative [24]. However, it is not clear whether animals, in general, or those domesticated primarily for production are able to distinguish between different human emotions based on facial expressions. To test this, we simultaneously presented goats with positive (happy) and negative (angry) images of unfamiliar human faces. Overall, goats differentiated the two sets of emotional expressions and preferred to approach happy faces first. In addition, they interacted first, more often and for longer duration with happy faces when they were presented on the right side rather than the left. This indicates that the potential for cross-species perception of emotions via human visual cues is far more widespread than previously believed [6,21].

We found that goats can distinguish between happy and angry images of the same person, indicating that they can visually differentiate human faces conveying different emotional valences. Domestication has been thought to enhance interspecies communication and emotion perception. While domestic non-companion animals have already demonstrated elaborate communicative skills when interacting with humans [32,33], research has generally not focused on the processing of emotions. However, studies on perception of human emotions in domestic species done with companion and working animals have shown, for example, that dogs are able to discriminate and categorize different emotional expressions of conspecifics and heterospecifics [23,24] and they avoid looking at angry human facial expressions compared to happy ones [38]. Instead of preferring happy faces, our goats could be avoiding the angry ones; the underlying mechanism is still to be sought. Our results provide evidence that a specific domestication history focused on working closely with humans or as companion animals is not a prerequisite for the ability to distinguish human emotions based on facial cues.

We found a lateralized response towards the human positive facial expressions. Goats significantly preferred to interact with the positive images when they were on the right side of the test arena, suggesting that there might be a differential engagement of the left-brain hemisphere for approaching happy faces (or an engagement of the right hemisphere for avoiding the negative images on the left side) [39,40]. This interaction of preference for positive-associated stimuli and side is in agreement with one major hypothesis on asymmetric hemispheric processing of emotional information in the mammalian brain: a right-hemisphere dominance for processing negative emotions, such as fear and aggression, and a left-hemisphere dominance for processing positive emotions [40,41]. Dogs, for example, turn left in response to aversive emotionally competent stimuli and thus process these with the right brain hemisphere [42,43], while they process positive social interactions and non-aversive olfactory cues with the left hemisphere [43,44]. In addition, horses show a preferential use of their right eye (right gaze bias, left-hemisphere processing) when looking at a human that has previously displayed a positive emotion towards them [26]. Moreover, horses show an orientation bias to the right side also for calls emitted from a familiar conspecific [45] and match human vocal and visual cues better when the familiar person is standing on their right side, indicating a left-hemisphere bias in recognizing familiar individuals [19]. Thus, the lateralized response that we found in goats could also be explained by differences in familiarity of the facial expression rather than valence, although both hypotheses are not mutually exclusive. Nevertheless, goats were still clearly able to distinguish the two types of faces.

Goats at our study site interact daily with familiar and unfamiliar humans. Thus, their amount of exposure to humans might have had an impact on their facial expression-processing abilities [32,33]. It is unlikely that the preferential interaction of our goats with happy faces is due to a specific conditioned response because we used unfamiliar faces that were never rewarded (or served as punishment) during the test, even though experience potentially plays a very important role in this cognitive processing. We hypothesize that goats generalized human facial features from previous positive (and negative) interactions with humans, which resulted in a preference for the happy images. This paves the way for future studies, such as testing animals with less frequent and/or less positive human interactions, which will provide additional insights into the development of goats’ ability to discriminate human facial expressions and its underlying mechanisms. These will answer the question of whether a specific background with humans (e.g. daily positive interactions) is necessary to enable goats to discriminate human emotional facial expressions or if goats, independent of their rearing background, are capable of reading subtle human facial cues.

## 5. Conclusion

We present the first evidence that goats can discriminate human facial expressions with different emotional information. Not only can they distinguish them, but they also generally prefer happy faces, regardless of the gender of the human faces or the sex of the goats. These findings suggest that the ability of animals to perceive human facial cues is not limited to those with a long history of domestication as companions, and therefore may be far more widespread than previously believed.

## Supplementary Material

ESM raw data

## Supplementary Material

ESM table 1

## Supplementary Material

ESM video

## Data accessibility

The data underlying this study are available from the electronic supplementary methods (electronic supplementary material, raw data).
